# Telepharmacy-based mobile health for diabetes care: models, benefits, and barriers-a scoping review

**DOI:** 10.1186/s12911-026-03736-2

**Published:** 2026-07-29

**Authors:** Amelia Rumi, Mila Karisma Fitriawati, Fakih Haikal, Risnandar, Ivan Surya Pradipta, Dika Pramita Destiani

**Affiliations:** 1 Doctoral Program in Pharmacy, Faculty of Pharmacy, Universitas Padjadjaran, Sumedang, West Java 45363 Indonesia; 2 Department of Pharmacology and Clinical Pharmacy, Faculty of Pharmacy, Universitas Padjadjaran, Sumedang, West Java 45363 Indonesia; 3 Research Center for Artificial Intelligence and Cybersecurity, National Research and Innovation Agency, Bandung, West Java 40135 Indonesia; 4 Drug Use and Pharmacoepidemiology Research Group, Center of Excellence in Higher Education for Pharmaceutical Care Innovation, Universitas Padjadjaran, Sumedang, West Java 45363 Indonesia; 5 Department of Pharmacy, Faculty of Mathematics and Natural Sciences, Universitas Tadulako, Central Sulawesi, Palu, 94118 Indonesia; 6 Computer Science, Nusa Putra University, Sukabumi, West Java 43152 Indonesia

**Keywords:** Diabetes mellitus, Telepharmacy, Mobile health, mHealth, Pharmacist

## Abstract

**Background:**

Diabetes mellitus (DM) requires continuous care to prevent complications. Telepharmacy delivered via mobile health (mHealth) applications offers a potential solution, particularly in resource-limited settings. However, the roles of pharmacists within such applications have not been comprehensively mapped.

**Objective:**

This scoping review aims to: (1) map existing models of telepharmacy-based mHealth applications for diabetes care, (2) describe the roles and functions of pharmacists within these models, and (3) synthesise the reported benefits and barriers associated with their implementation.

**Methods:**

The review followed the Arksey & O’Malley framework and PRISMA-ScR guidelines. We searched PubMed, Scopus, and Web of Science from inception to 20 May 2026. Studies were eligible if they involved pharmacists, used a mobile application for telepharmacy, and targeted any type of diabetes. Data extraction covered study characteristics, pharmacist roles, application features, benefits, and barriers.

**Results:**

Of 227 records, 6 studies (632 patients) met the inclusion criteria. Studies were conducted in Malaysia, China, Japan, Pakistan, Thailand, and Indonesia covering type 1, type 2, elderly type 2, and gestational diabetes. Pharmacist roles included digital coach, educational facilitator, clinical consultant, and therapy monitor. Key application features comprised electronic medical records, counselling dashboards, adherence and quality of life assessments, medication reminders, and lifestyle monitoring. Reported benefits included improved glycaemic control (HbA1c reduction up to 0.79%), blood pressure, medication adherence, and quality of life (*p* < 0.05 where reported). Barriers included low digital literacy, technical issues (data synchronisation), waning long-term motivation, and economic constraints (subscription costs).

**Conclusion:**

Telepharmacy-based mHealth applications with active pharmacist involvement may contribute to improving comprehensive diabetes management. However, evidence is currently limited. Future applications should prioritise user-centred design, sustainable engagement strategies, and supportive policy frameworks.

**Clinical trial number:**

Not applicable.

**Supplementary Information:**

The online version contains supplementary material available at https://doi.org/10.1186/s12911-026-03736-2.

## Background

Diabetes mellitus is a chronic metabolic disorder characterised by persistent hyperglycaemia, requiring lifelong management to prevent macrovascular and microvascular complications. In 2024, the global prevalence reached 588.7 million, with 3.4 million deaths; projections estimate 852.5 million by 2050 [1]. The increasing prevalence of diabetes presents significant obstacles in providing adequate healthcare services [2]. Effective diabetes management involves lifestyle modification, pharmacological therapy, and regular glucose monitoring [3]. Despite advances in treatment, many patients face barriers to accessing optimal care, including transportation difficulties, inadequate healthcare infrastructure, and geographical limitations, particularly in rural or resource-constrained settings [4]. These obstacles underscore the need for sustainable, adaptable digital health solutions. Telepharmacy has been developed as an innovative digital healthcare solution, enhancing the reach and effectiveness of remote medical services via mobile platforms [5]. Pharmacists play a crucial role in diabetes management in countries with inadequate infrastructure, including a lack of pharmaceutical care services, particularly in the continuous monitoring and evaluation of therapeutic outcomes. Compared to other healthcare professionals, pharmacists play a strategic role in interacting directly with patients [6, 7].

Telepharmacy - the provision of pharmaceutical care through telecommunications has emerged as an innovative strategy to extend the reach of pharmacy services [8]. Although the integration of information and communication technology (ICT) into pharmacy practice has progressed over the past decade, its effects on clinical outcomes and medication adherence have not been consistently established [9]. Pharmacists play a critical role in diabetes management because of their direct and frequent interaction with patients, particularly in medication review, adherence monitoring, and therapeutic outcome evaluation. During the COVID-19 pandemic, telepharmacy proved essential for maintaining continuity of care when in-person consultations were not possible [10].

The first telepharmacy model was introduced in the early 2000s in countries such as the United States, Canada, and Australia. The use grew significantly during the COVID-19 pandemic [11]. This innovation has proven to be particularly crucial in areas with limited resources [12]. In the United States, it allows pharmacists to assess prescriptions and manage pharmacy operations using video conferencing. Pharmacists in Canada are permitted to provide services and charge fees for virtual care, including online consultations. National directives from pharmaceutical associations have been implemented across Europe to provide remote consultations by telephone, video, and email. In the United Kingdom, these professionals use an electronic documentation system to carry out online consultations and oversee medication deliveries. Overall, telepharmacy has enabled pharmacists to sustain healthcare services while decreasing administrative burdens [13]. In this era, studies have reported that pharmacists’ roles in telepharmacy have expanded through mobile pharmacy applications, encompassing medication information provision, patient education, therapeutic follow-up, adverse drug reaction detection, and pharmacist–patient interactions [14].

Mobile health (mHealth), defined by the World Health Organization as the use of mobile technologies to support medical and public health services, has further expanded the potential of telepharmacy [15]. Wearable devices and smartphone applications enable real-time data collection, personalised feedback, and remote monitoring. When integrated with pharmacist-led interventions, mHealth can promote proactive, data-driven diabetes care [16]. mHealth, including telepharmacy, has the potential to improve glycemic control and increase medication adherence [15, 17].

A significant limitation of current telepharmacy systems and pharmacist-led digital interventions in diabetes management is the lack of personalization [7]. This limitation represents a significant drawback, as studies show that personalized Mobile Health Programs (MHPs), particularly those augmented by AI and tailored behavioral data, produce better clinical outcomes [16]. A major challenge in implementing such systems is the protection of patient data, specifically in compliance with regulations, such as the General Data Protection Regulation (GDPR). Integrating these services can substantially reduce the time required for treatment assessments [18]. Several previous reviews have examined digital health interventions provided by pharmacists for individuals with diabetes to over limited personalisation [5, 7] or telepharmacy in general [14], but none have comprehensively mapped the models of telepharmacy-based mHealth applications, the specific roles and functions of pharmacists within these models, or the reported benefits and barriers to their implementation – all in the context of diabetes care. This gap is particularly important because understanding existing models is essential for developing future, scalable, and sustainable applications. Therefore, this scoping review aims to:

1. Map existing models of telepharmacy-based mHealth applications in diabetes care,
2. Describe the roles and functions of pharmacists within these models,
3. Synthesise the benefits and barriers associated with their implementation.

Given this context, a comprehensive mapping of existing telepharmacy-based mobile application models, specifically those involving pharmacists in diabetes care, is lacking. Therefore, this review aims to map existing models of telepharmacy-based mobile health applications in diabetes care, to describe the roles and functions of pharmacists within these models, and synthesize the reported benefits and barriers associated with their implementation.

## Materials and methods

### Study design

This scoping review followed the framework developed by Arksey & O’Malley (2005) [19], which comprises five stages: (1) identifying the research question, (2) identifying relevant studies, (3) study selection, (4) charting the data, and (5) collating, summarising, and reporting the results. We report the findings in accordance with the Preferred Reporting Items for Systematic Reviews and Meta-Analyses extension for Scoping Reviews (PRISMA-ScR). The completed PRISMA-ScR checklist is provided as Supplementary File 2. The scope review was selected for exploration and analysis to map the models, benefits, and barriers associated with pharmacist-led features in diabetes care practices.

### Search strategy

We systematically searched three electronic databases: PubMed, Scopus, and Web of Science. The search was performed from database inception to 20 May 2026 (the original search was up to 31 May 2025; we repeated the search on 20 May 2026 to update the evidence). The search strategy combined terms related to telepharmacy, mobile health, diabetes, and pharmacists. Detailed search strings are provided in Appendix 1. No publication date or language limits were applied during the database search. Nevertheless, the review ultimately included only English-language peer-reviewed articles while grey literature was excluded.

### Eligibility criteria

We defined eligibility using the Population-Concept-Context (PCC) framework:

**Table:** 

PCC element	Inclusion criteria	Exclusion criteria
Population	Pharmacists (any practice setting).	No pharmacist involvement was identified in the mobile application.
Concept	Telepharmacy delivered via a mobile application (mHealth) including smartphone or tablet apps.	Not primarily focused on mobile applications (e.g., Text-only messaging, regular phone calls, email, SMS, websites, non-mobile software, or standalone hardware), home care program, mobile app focused on drug interaction, not related digital health.
Context 1	Diabetes mellitus (any type: T1DM, T2DM, gestational, or elderly).	Other diseases without diabetes focus.
Context 2	Barrier associated with difficulties in the adoption, usability, or effective implementation of a mobile application.	-

The inclusion criteria of primary peer-reviewed research (RCT, quasi-experimental, before-after study, cohort, or mix-method study). Exclusion criteria included survey, proceeding, reports, editorials, opinion pieces, conference abstracts, preprints, dissertations, study protocols, non-English articles. Gray literature was excluded from this scoping review to maintain methodological rigor and ensure that all included evidence had undergone peer-reviewed processes, thereby supporting the consistency and reliability of the synthesized findings. We acknowledge this decision as a potential limitation (see Strengths and Limitations).

### Selection process

Search results were exported to Rayyan.ai [20] for duplicate removal and screening. Three researchers (AR, MKF, and FH) independently screened titles and abstracts against the eligibility criteria. Potentially eligible studies subsequently underwent full-text assessment. Discrepancies regarding study eligibility were resolved through iterative discussion and consensus with DPD. To strengthen methodological rigor and minimize subjective bias, investigator triangulation was applied through independent cross-checking by DPD, ISP, and RIS.

### Data extraction

Data extraction were independently performed by AR using a standardized data-charting form. A consensus-based approach was employed to address uncertainties or discrepancies during the extraction process. To enhance credibility and consistency of the extracted findings, investigator triangulation was subsequently conducted by DPD, ISP, and RIS through independent verification and cross-checking procedures. The following items were extracted: (a) Bibliographic information (author, year, country); (b) Study design and setting; (c) Patient population (type of diabetes, sample size, age); (d) Intervention details (name of application, platform, duration); (e) Pharmacist roles and functions; (f) Key application features; (g) Reported benefits (clinical, behavioural, psychological); and (h) Reported barriers or disadvantages.

## Results

### Overview of the evidence base

**Fig. 1:**
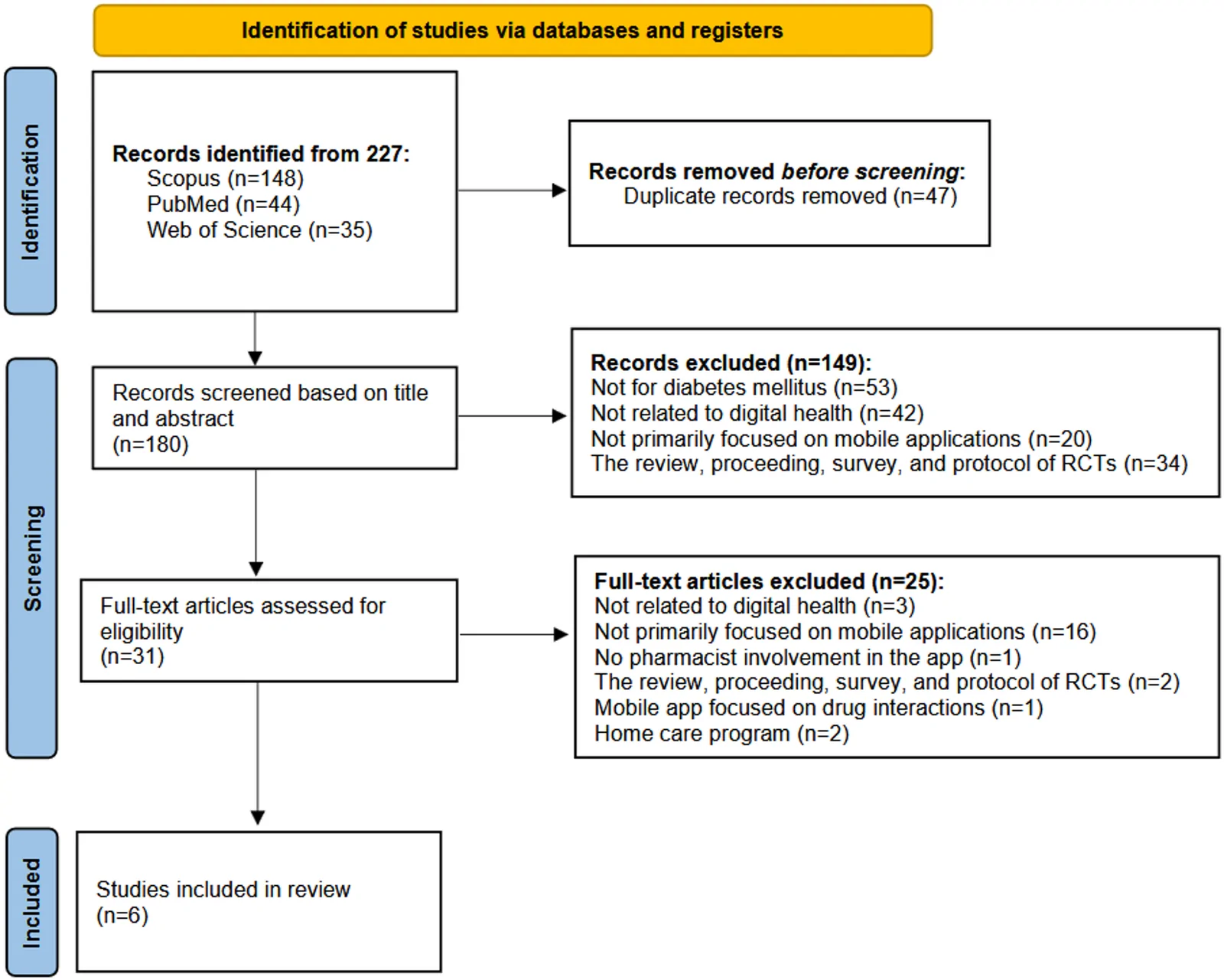
Flow diagram of PRISMA-scoping review (ScR)

Our search yielded 227 records. Following duplicate removal and screening based on the eligiblity criteria, six studies were ultimately included in this scoping review, as presented in the PRISMA-ScR flow diagram in Fig. 1. The small number of studies included reflects the focused scope and predefined eligibility criteria of this review. Although many records addressed mHealth, telemedicine, or diabetes self-management more generally, only a limited number specifically examined pharmacist-led telepharmacy-based mobile interventions and reported outcomes relevant to the objectives of this scoping review. These studies were conducted in six countries: Malaysia, China, Japan, Pakistan, Thailand, and Indonesia. Study designs included randomised controlled trials (RCTs), cluster RCTs, and quasi-experimental studies. A total of 632 patients were enrolled, covering type 1 diabetes mellitus (T1DM), type 2 diabetes mellitus (T2DM), T2DM in older adults, and gestational diabetes mellitus (GDM). Intervention durations ranged from 6 weeks to 12 months (as summarized in Table 1) [21–26].

**Table 1:** Characteristics of included studies (overview)

No.	Author (year)	Country	Study design	Diabetes type	Sample size (*n*)	Intervention duration
1	Sze et al. (2023) [21]	Japan	Quasi- experimental	T2DM	33	12 weeks
2	Hussain et al. (2024) [22]	Pakistan	RCT	T1DM (adolescents)	112	12 months
3	Johari et al. (2024) [23]	Malaysia	Quasi- experimental	T2DM	111	6 months
4	Poonprapai et al. (2022) [24]	Thailand	RCT	Elderly T2DM	157	9 months
5	Zhuo et al. (2022) [25]	China	RCT	Gestational DM	119	12 weeks postpartum
6	Rindarwati et al. (2025) [26]	Indonesia	Quasi- experimental	T2DM	100	24 weeks

Note: RCT Randomized Controlled Trial, DM diabetes mellitus, T1DM Type 1 Diabetes Mellitus, T2DM Type 2 Diabetes Mellitus

### Model of use of telepharmacy-based mobile health for DM

In all six studies, pharmacists were integral members of a multidisciplinary team that included physicians, nutritionists, and nurses. The models varied in their specific implementation but shared common features: real-time data monitoring, patient education, medication management, and lifestyle support [21–25]. Sze et al. (2023) [21] described a model where pharmacists acted as digital coaches. They encouraged optimal app use, provided lifestyle advice, and helped patients interpret health data (step counts, blood pressure, weight). Pharmacists collaborated with patients to set daily physical activity goals and develop problem-solving strategies. Hussain et al. (2024) [22] presented a model in which pharmacists served as educational facilitators. They supervised self-monitoring of blood glucose (SMBG), taught patients to understand the therapeutic implications of their data, and enabled timely detection of hypo- or hyperglycaemia. Johari et al. (2024) [23] have implemented the SugO365 program using the Health2Sync application that allows pharmacists, nutritionists and dietitians to provide personalized education and support through a shared digital dashboard based on patients’ glucose monitoring data supported by face-to-face educational sessions and quarterly webinars to improve diabetes self-management.

Poonprapai et al. (2022) [24] developed a family-support model. Pharmacists educated family caregivers using interactive infographics and quizzes, sent automated medication reminders, and enabled bidirectional communication between caregivers and pharmacists for personalised feedback. The study of Zhuo et al. (2022) [25] created the Continuing Medical Care (CMC) app. Clinical pharmacists posted educational resources (videos, articles) on insulin storage, injection technique, and hypoglycaemia management. They also communicated with patients in real time, adjusted insulin doses (in consultation with endocrinologists), and monitored adherence. Then, study of Rindarwati et al. (2025) created the Sahabat DM, pharmacists contributed to educational content development, patient counseling, medication adherence support, real-time consultation, patient engagement monitoring, follow-up counseling, lifestyle modification support, medication side-effect reporting, and user training within the Sahabat DM mobile health application [26].

Table 2 summarizes the clinical outcomes, roles of pharmacist, and implementation challenges described in the included studies, and Table 3 maps the integration of mobile application features with pharmacist functions supporting comprehensive telepharmacy services. Beyond the usual pharmaceutical management, the pharmacist’s role depended on the model of intervention, such as digital coaching, patient and family education, clinical consultation, therapeutic monitoring, lifestyle counselling, and adherence support. Pharmaceutical services given via mobile applications were examined, regulated and feedback was provided by pharmacists before and during implementation [21, 22, 25]. Additionally, pharmacists assisted family caregivers by offering information and reminders to support effective use of the applications and to encourage self-management behaviors [24, 26]. These services were supplemented by physicians and nutritionists who remotely monitored the patient data and provided condition-specific or nutritional coaching during the treatment [23]. Together, our findings demonstrate the potential of telepharmacy-based mHealth interventions to integrate pharmaceutical care into multidisciplinary diabetes management, targeting clinical and behavioral aspects of care in varied healthcare settings.

**Table 2:** Outcomes and pharmacist roles

No.	Study (Years)	Key outcomes (*p* < 0.05)	Pharmacist role	Disadvantages
1	Sze et al. (2023) [21]	Decreased BMI, BP systolic/diastolic, HbA1c, and FPG.	Digital coach, lifestyle educator	iOS-only and data synchronisation issues
2	Hussain et al. (2024) [22]	Decreased HbA1c, ketoacidosis, and hypoglycaemic episodes.	Educational facilitator, real-time support	Limited app evaluation, language constraints
3	Johari et al. (2024) [23]	Decreased weight, BMI, RBG, HbA1c, and improved quality of life.	Lifestyle educator	Subscription cost and economic access
4	Poonprapai et al. (2022) [24]	Decreased BP systolic/diastolic, HbA1c, and increased adherence.	Patient and family educator, therapy monitor	Insulin therapy patients excluded
5	Zhuo et al. (2022) [25]	Decreased FPG, HbA1c, hypoglycaemia, and increased adherence treatment.	Clinical consultant, dose adjustment, educator	Educational gap and manual data entry
6	Rindarwati et al. (2025) [26]	Decreased HbA1c.	Health facilitator and counselor medication adherence and lifestyle medication	Limited access to continous education and low digital literacy

Note: HbA1c Glycosylated hemoglobin, RBG Random Blood Glucose, FPG fasting plasma glucose, BP blood pressure, BMI body mass index, SMBG self-monitoring blood glucose

**Table 3:** Integrated features and pharmacist roles

Feature	Pharmacist role	Function	Reference
Electronic medical records	Pharmacist review and suggestions	Real-time drug interaction / adjustment	[21, 25]
Dashboard live chat/video	Virtual one-on-one communication	Pharmacist-nutritionist-patient collaboration	[23, 26]
Assessment tools	Follow-up and tracking	Online adherence, quality of life, diabetes distress scales	[22, 23, 25]
Medication reminders	Adherence monitoring	Push notifications for medications	[22, 24–26]
Clinical data monitoring	Monitoring clinical parameters	Glucose, blood pressure, weight tracking	[21, 23, 25, 26]
Educational articles/videos	Patient education	Medication, injection technique, hypoglycaemia	[22, 25, 26]
Lifestyle modification	Lifestyle education	Diet management	[23]
Physical activity tracking	Increased physical activity	Step count, goal setting (physical activity)	[21, 26]
Medication side-effect reporting	Pharmacists assess reported symptoms, identify potential drug-related problems, provide recommendations, and monitor medication safety	Enables patients to report adverse drug reactions	[26]

The aforementioned models showed that the integration of mobile applications with the proactive involvement of pharmacists created a comprehensive approach to diabetes management. mHealth applications functioned as tools for data documentation, as well as platforms for education, communication, and continuous therapeutic oversight. The active engagement of pharmacists enhanced patient adherence to treatment, facilitated the early detection of issues, and promoted the optimization of patient outcomes.

### Benefits of telepharmacy-based mHealth

All six studies reported significant clinical benefits. Glycaemic control improved across all diabetes types, with reductions in HbA1c ranging from 0.5% to 0.79% (*p* < 0.05) [21–26]. Sze et al. (2023) also reported decreased systolic and diastolic blood pressure and increased daily step count [21]. Hussain et al. (2024) noted reductions in ketoacidosis and hypoglycaemic episodes [22]. Johari et al. (2024) found improvements in both physical and mental quality of life [23]. Poonprapai et al. (2022) and Zhuo et al. (2022) reported significant increases in medication adherence [24, 25]. Then, Rindarwati et al. (2025) reported pharmacists provided real-time, patient-centered consultations through integrated chat and call features in the application. Patients were able to receive timely advice regarding medication use, side effects, lifestyle modification, and other treatment-related concerns. Additional benefits included enhanced patient empowerment (particularly among adolescents with T1DM), culturally relevant education (Urdu language app), and the feasibility of hybrid (online/offline) models in pharmacy settings. The family-support model proved cost-effective and scalable.

As a result, the use of tailored mHealth applications by pharmacists proved to be effective in optimizing treatment, supporting self-care, and promoting a more independent, sustainable, and integrated approach to managing diabetes.

### Barriers to telepharmacy-based mHealth

Several barriers were identified across studies:

- Technical issues: Data synchronisation failures between wearable devices and apps [21] and manual data entry requirements [25].
- Digital literacy: Patients required education on app usage; older adults faced particular difficulties [26].
- Motivation and consistency: Long-term adherence to app use declined, especially when no immediate feedback was provided [23, 26].
- Economic constraints: Subscription-based models by Johari et al. (2024) created socioeconomic inequity [23], favouring patients with higher incomes.
- Language and content limitations: Only apps supporting Urdu were included in one study [22], excluding those with more advanced features.
- Study design restrictions: Insulin-treated patients were excluded in Poonprapai et al. (2022) [24], limiting generalisability.

## Discussion

This scoping review found that pharmacists were consistently involved as key members of multidisciplinary teams delivering telepharmacy-supported mHealth interventions for diabetes care [21–26]. Rather than serving only as medication providers, pharmacists participated in a wide range of activities that included patient education, medication monitoring, clinical consultation, and digital coaching [21–26]. Although the interventions differed across studies, several common features emerged. Mobile applications were used not only to collect patient data but also to maintain ongoing communication among patients and healthcare professionals. Through these platforms, pharmacists were able to review electronic medical records, monitor SMBG results, provide individualised feedback, educate family caregivers, and collaborate with endocrinologists when medication adjustments were required [21–23, 25, 26]. Collectively, these results indicate that digital technologies are enabling pharmacists to take a more active role in chronic disease management, extending their contribution past traditional dispensing activities.

The roles identified in this review are generally consistent with those described in the wider telepharmacy and mHealth literature. Previous studies have shown that pharmacists can contribute to medication therapy management, remote patient monitoring, and coordinated care within collaborative practice models, frequently resulting in considerable improvements in glycaemic outcomes [27–29]. Similar patterns were observed in the present review, where all six studies found significant reductions in HbA1c despite being conducted in different healthcare settings and involving diverse diabetes populations [21–26]. Several examples show how mobile health technologies can translate established pharmaceutical care principles into routine practice. These include the use of wearable devices to support digital coaching [21], educational programmes for family caregivers delivered through interactive infographics [24], and pharmacist-led insulin titration supported by real-time dashboard systems [25] While such functions have been discussed in previous literature, the included studies present practical examples of their implementation. Existing evidence also suggests that pharmacist-led telemonitoring programmes can achieve HbA1c improvements comparable to, or in some cases greater than, those observed with usual care, particularly when pharmacists work alongside health coaches and primary care providers [29–32]. The findings of this review therefore add further support to the growing evidence base for team-based telepharmacy interventions. In addition, successful implementation of hybrid online–offline services and culturally adapted applications, including Urdu-language platforms, indicates that these methods may help reach populations that are traditionally underserved by healthcare systems [22, 23].

The benefits reported across the included studies were not limited to glycaemic outcomes. Several studies documented improvements in blood pressure, body mass index, medication adherence, and quality of life [21, 23–25]. In studies where pharmacists provided real-time support for SMBG, reductions in hypoglycaemic events and episodes of ketoacidosis were also observed [24, 25]. Other positive outcomes included greater patient empowerment among adolescents living with type 1 diabetes mellitus (T1DM) and increased physical activity achieved through goal-setting features and step-count monitoring [21, 26]. These findings are consistent with previous telehealth and mHealth research demonstrating improvements in patient activation, self-management confidence, treatment adherence, and satisfaction with pharmacist-led services [30, 33–35]. Many of the mobile applications incorporated features such as medication reminders, educational content, adverse effect reporting tools, and lifestyle management resources [22, 24–26]. By combining these functions within a single platform, pharmacists were able to deliver care that addressed both clinical and behavioural aspects of diabetes management. Continuous monitoring of patient-reported outcomes and clinical parameters also allowed pharmacists to identify problems early and provide timely recommendations, which may contribute to improved adherence, safer medication use, and more patient-centred care [31, 35, 36].

Despite the encouraging findings, several challenges were identified. Technical limitations were reported in some studies, including problems with synchronising wearable devices and mobile applications as well as dependence on manual data entry, both of which affected data quality and user experience [21, 25]. Digital literacy was another recurring issue. Older adults and individuals with limited experience using mobile technologies often required additional training and support before they could engage effectively with the interventions [26]. Sustaining long-term engagement also appeared challenging. In some studies, patient motivation declined over time, particularly when applications did not provide immediate or meaningful feedback [23, 26]. Financial barriers were evident as well. Subscription-based services may restrict participation among lower-income populations, potentially widening existing health inequalities [23]. Some interventions were available only in specific languages or required a certain level of technological proficiency, which may have excluded groups that could otherwise benefit from these services [22]. Furthermore, eligibility restrictions within individual studies, such as the exclusion of insulin-treated patients, reduced the extent to which findings could be applied to broader diabetes populations [24]. These observations reflect concerns that have been raised elsewhere in the telepharmacy literature, including issues related to privacy and data security, uneven access to internet connectivity and digital devices, uncertainty regarding reimbursement mechanisms, regulatory challenges, and the additional workload that may be placed on pharmacists [30, 33, 35, 37–39]. Taken together, the evidence suggests that although telepharmacy-supported mHealth interventions are promising, wider implementation will require solutions that address technological, organisational, and policy-related barriers.

### Future research directions

Several research gaps emerge from this review. Rigorous randomized controlled trials with standardized clinical, behavioral, and economic outcome measures are needed to strengthen the evidence base and support policy decisions [38, 40]. Greater consistency in outcome reporting across studies would facilitate future meta-analysis and enable more robust comparisons between interventions.

Implementation science approaches should be applied to understand how digital literacy interventions, patient engagement strategies, and equitable access mechanisms can be embedded into telepharmacy programs from the outset [41]. Research is also needed to identify practical approaches for maintaining long-term patient motivation and ensuring that the benefits of mHealth interventions are accessible across different socioeconomic groups, particularly in low- and middle-income countries.

The integration of artificial intelligence and advanced analytics into pharmacist-led platforms warrants dedicated investigation, particularly for pharmacovigilance and early adverse event detection [41]. Additionally, evidence regarding cost-effectiveness and sustainable reimbursement strategies remains largely absent from the current literature but will be essential if telepharmacy services are to become a routine component of diabetes care [31]. Addressing these priorities may help move the field beyond small-scale demonstrations and support the development of telepharmacy models that are effective, equitable, and sustainable in real-world practice.

### Future implications

The evidence gathered in this review signals something broader than the sum of its clinical outcomes, it points toward a fundamental repositioning of the pharmacist within the healthcare system. As digital health infrastructure continues to expand globally, pharmacists are increasingly well-placed to serve as the connective tissue between patients, data, and multidisciplinary care teams. The models identified here demonstrate that this role is not aspirational; it is already being enacted in diverse settings, from Malaysian primary care to Indonesian community pharmacy and Pakistani adolescent diabetes clinics. In health systems where physician shortages and geographic barriers limit access to specialist care, pharmacist-led mHealth interventions offer a pragmatic and scalable pathway to closing treatment gaps a consideration of particular relevance for low- and middle-income countries (LMICs) such as Indonesia.

From a policy perspective, these findings make a compelling case for formally recognizing and resourcing the expanded clinical role of pharmacists in digital health delivery. Workforce development frameworks will need to evolve to equip pharmacists with competencies in data interpretation, digital coaching, and remote consultation. Regulatory bodies and payers must also engage with the question of how telepharmacy services are defined, credentialled, and reimbursed because without structural support, even the most effective models risk remaining confined to pilot studies. Looking further ahead, the convergence of mobile health platforms, artificial intelligence, and pharmacist clinical expertise holds genuine promise for transforming diabetes management into a more proactive, personalized, and continuously monitored endeavor. Pharmacists, as medication and patient safety experts embedded in communities, are uniquely positioned to lead that transformation.

### Strength and limitations

Strengths: This is the first scoping review to comprehensively map pharmacist-integrated mHealth models for diabetes care. We followed rigorous scoping review methodology (Arksey & O’Malley, PRISMA-ScR) and updated the search to 20 May 2026.

Limitations:

- Only six studies were available, limiting generalisability.
- We excluded grey literature. This was a deliberate choice to focus on peer-reviewed, fully reported interventions, but it may have omitted relevant ongoing or unpublished work.
- As a scoping review, we did not critically appraise study quality or synthesise effect sizes; conclusions about efficacy are therefore preliminary.
- The search was restricted to three databases and English-language articles, possibly missing some regional or non-English publications.

## Conclusion

In conclusion, this scoping review synthesises evidence on telepharmacy-based mHealth models for diabetes care. Pharmacists can effectively function as digital care coordinators and educators through features such as electronic medical records, counselling dashboards, quality of life and adherence assessment tools, medication reminders, lifestyle monitoring, and clinical data monitoring. Their involvement has been shown to provide multidimensional benefits: improved glycaemic control, blood pressure, medication adherence, and quality of life. However, the evidence base is currently limited, and implementation faces barriers including low digital literacy, technical challenges, waning motivation, and economic constraints. Future efforts must focus on creating sustainable, user-centred, and policy-supported models that leverage pharmacists’ unique expertise within the digital health ecosystem.

## Supplementary Information

Below is the link to the electronic supplementary material.

Supplementary Material 1: Appendix 1: Detailed search strings (PubMed, Scopus, Web of Science), and Supplementary File 2: PRISMA-ScR checklist

## Data Availability

All data generated or analysed during this study are included in this published article and its supplementaryinformation files. The search strategy and PRISMA-ScR checklist are provided as supplementary materials. Noprimary data were collected for this scoping review.

## Abbreviations

DM: Diabetes Mellitus
T1DM: Type 1 Diabetes Mellitus
T2DM: Type 2 Diabetes Mellitus
GDM: Gestational Diabetes Mellitus
mHealth: Mobile Health
ICT: Information and Communication Technology
SMBG: Self-Monitoring of Blood Glucose
HbA1c: Glycosylated Hemoglobin
RCT: Randomized Controlled Trial
PRISMA-ScR: Preferred Reporting Items for Systematic Reviews and Meta-Analyses extension for Scoping Reviews
PCC: Population-Concept-Context
GDPR: General Data Protection Regulation
