# Supplementary material for: Telepharmacy-based mobile health for diabetes care: models, benefits, and barriers-a scoping review
Source: BMC Med Inform Decis Mak. 2026 Jul 29;26:431. doi: 10.1186/s12911-026-03736-2 (PMC13700386; doi:10.1186/s12911-026-03736-2)
Supplement: Supplementary file 1 [file 12911_2026_3736_MOESM1_ESM.docx]

**Appendix I. Search Strategy**

| **Database** | **Concept** | **Search Term** | **Result** | **Date** |
| --- | --- | --- | --- | --- |
| **PubMed** | Population | ((((((((((Pharmacist) OR Pharmacist[Title/Abstract]) OR ("clinical pharmacist"[Title/Abstract])) OR ("professional pharmaceutical care services"[Title/Abstract])) OR ("pharmaceutical care"[Title/Abstract])) OR ("hospital pharmacy service"[Title/Abstract])) OR ("community pharmacy service"[Title/Abstract])) | 62,714 | 20/05/2026 |
|  | Concept | ((((((((((((((((((((((Telepharmacy) OR (telemedicine[Title/Abstract])) OR (telehealth[Title/Abstract])) OR ("digital health"[Title/Abstract])) OR (eHealth[Title/Abstract])) OR (mHealth[Title/Abstract])) OR ("cell phone"[Title/Abstract])) OR ("mobile phone"[Title/Abstract])) OR (smartphone[Title/Abstract])) OR ("mobile health"[Title/Abstract])) OR ("mobile application"[Title/Abstract])) OR (applications[Title/Abstract])) OR (app[Title/Abstract])) OR (apps[Title/Abstract])) OR ("information and communication technologies"[Title/Abstract])) OR (software[Title/Abstract])) OR ("technological tools"[Title/Abstract])) OR ("intelligent medication manager"[Title/Abstract])) OR (Telecounselling[Title/Abstract])) OR (telemonitoring[Title/Abstract])) OR ("text-based reminder"[Title/Abstract])) OR ("mobile reminder"[Title/Abstract]) | 1,360,277 | 20/05/2026 |
|  | Context 1 | (((((("Diabetes mellitus") OR (DM[Title/Abstract])) OR ("type 2 diabetes mellitus"[Title/Abstract])) OR (T2DM[Title/Abstract])) OR ("type 1 diabetes mellitus"[Title/Abstract])) OR (diabetic[Title/Abstract]) | 779,611 | 20/05/2026 |
|  | Context 2 | (((((Barrier) OR (barriers[Title/Abstract])) OR (challenge[Title/Abstract])) OR (challenges[Title/Abstract])) OR (concern[Title/Abstract]) | 2,065,832 | 20/05/2026 |
|  | All Concept | ((((((((Pharmacist) OR ("clinical pharmacist"[Title/Abstract])) OR ("professional pharmaceutical care services"[Title/Abstract])) OR ("pharmaceutical care"[Title/Abstract])) OR ("hospital pharmacy service"[Title/Abstract])) OR ("community pharmacy service"[Title/Abstract])) AND (((((((((((((((((((((((Telepharmacy) OR (telemedicine[Title/Abstract])) OR (telehealth[Title/Abstract])) OR ("digital health"[Title/Abstract])) OR (eHealth[Title/Abstract])) OR (mHealth[Title/Abstract])) OR ("cell phone"[Title/Abstract])) OR ("mobile phone"[Title/Abstract])) OR (smartphone[Title/Abstract])) OR ("mobile health"[Title/Abstract])) OR ("mobile application"[Title/Abstract])) OR (applications[Title/Abstract])) OR (app[Title/Abstract])) OR (apps[Title/Abstract])) OR ("information and communication technologies"[Title/Abstract])) OR (software[Title/Abstract])) OR ("technological tools"[Title/Abstract])) OR ("intelligent medication manager"[Title/Abstract])) OR (Telecounselling[Title/Abstract])) OR (telemonitoring[Title/Abstract])) OR ("text-based reminder"[Title/Abstract])) OR ("mobile reminder"[Title/Abstract]))) AND (((((("Diabetes mellitus") OR (DM[Title/Abstract])) OR ("type 2 diabetes mellitus"[Title/Abstract])) OR (T2DM[Title/Abstract])) OR ("type 1 diabetes mellitus"[Title/Abstract])) OR (diabetic[Title/Abstract]))) AND (((((Barrier) OR (barriers[Title/Abstract])) OR (challenge[Title/Abstract])) OR (challenges[Title/Abstract])) OR (concern[Title/Abstract])) | 44 | 20/05/2026 |
| **Scopus** | Population | TITLE-ABS-KEY ( pharmacist OR "clinical pharmacist" OR "professional pharmaceutical care services" OR "pharmaceutical care" OR "hospital pharmacy service" OR "community pharmacy service" ) | 142,892 | 20/05/2026 |
|  | Concept | TITLE-ABS-KEY (tele-pharmacy OR telemedicine OR "digital health" OR e-health OR m-health OR "Cell Phone" OR "Mobile Phone" OR "mobile health" OR "mobile application" OR applications OR app OR apps OR "Information and communication technologies" OR software OR "technological tools" OR "intelligent medication manager " OR tele-counseling OR tele-monitoring OR "text-based reminder" OR "mobile reminder") | 12,189,859 | 20/05/2026 |
|  | Context 1 | TITLE-ABS-KEY ("Diabetes mellitus" OR "type 2 diabetes mellitus" OR "type 1 diabetes mellitus" OR diabetic) | 1,317,641 | 20/05/2026 |
|  | Context 2 | TITLE-ABS-KEY (barrier OR barriers OR challenge OR challenges OR concern) | 6,686,000 | 20/05/2026 |
|  | All Concept | TITLE-ABS-KEY ( pharmacist OR "clinical pharmacist" OR "professional pharmaceutical care services" OR "pharmaceutical care" OR "hospital pharmacy service" OR "community pharmacy service" ) AND TITLE-ABS-KEY ( tele-pharmacy OR telemedicine OR "digital health" OR e-health OR m-health OR "Cell Phone" OR "Mobile Phone" OR "mobile health" OR "mobile application" OR applications OR app OR apps OR "Information and communication technologies" OR software OR "technological tools" OR "intelligent medication manager " OR tele-counseling OR tele-monitoring OR "text-based reminder" OR "mobile reminder" ) AND TITLE-ABS-KEY ( "Diabetes mellitus" OR "type 2 diabetes mellitus" OR "type 1 diabetes mellitus" OR diabetic ) AND TITLE-ABS-KEY ( barrier OR barriers OR challenge OR challenges OR concern ) | 148 | 20/05/2026 |
| Web of Science | Population | TS=( ( pharmacist* OR "clinical pharmacist*" OR "pharmaceutical care" OR "hospital pharmacy service*" OR "community pharmacy service*" ) | 47,867 | 20/05/2026 |
|  | Concept | TTS=( ( telepharmacy OR telemedicine OR telehealth OR "digital health" OR eHealth OR mHealth OR "mobile health" OR "mobile application*" OR smartphone* OR telemonitoring OR telecounselling OR "text-based reminder*" OR "mobile reminder*" ) | 140,556 | 20/05/2026 |
|  | Context | TS=( ( "diabetes mellitus" OR "T2DM" OR "type 2 diabetes mellitus" OR "type 1 diabetes mellitus" ) ) | 282,159 | 20/05/2026 |
|  | All Concept | TS=( ( pharmacist* OR "clinical pharmacist*" OR "pharmaceutical care" OR "hospital pharmacy service*" OR "community pharmacy service*" ) AND ( telepharmacy OR telemedicine OR telehealth OR "digital health" OR eHealth OR mHealth OR "mobile health" OR "mobile application*" OR smartphone* OR telemonitoring OR telecounselling OR "text-based reminder*" OR "mobile reminder*" ) AND ( "diabetes mellitus" OR "T2DM" OR "type 2 diabetes mellitus" OR "type 1 diabetes mellitus" ) ) | 35 | 20/05/2026 |
